# Microwave ablation for the control of bleeding from disintegrated mammary tumours in two dogs

**DOI:** 10.1002/vms3.1089

**Published:** 2023-02-06

**Authors:** Yuta Kawamura, Hiroki Itou, Akitomo Kida, Hiroki Sunakawa, Kenji Kawamura

**Affiliations:** ^1^ Kawamura Animal Hospital Niigata Niigata Japan; ^2^ Department of Radiology, Division of Diagnostic Radiology, Faculty of Medicine Yamagata University Yamagata Japan

**Keywords:** breeding, cancer, dog, haemostasis, mammary gland

## Abstract

A 16‐year‐old intact female Miniature Dachshund (dog 1) and a 13‐year‐old intact female American Cocker Spaniel (dog 2) presented with a chief complaint of bleeding from a mammary gland tumour ulceration. Dog 1 was transferred to hospital from a local hospital in a haemorrhagic shock state with uncontrolled continuous bleeding. Thoracic radiographs revealed multiple nodular shadows suspected to be pulmonary metastasis. Dog 2 presented with intermittent bleeding from a mass lesion in the right fifth mammary gland. Due to high anaesthetic risk secondary to severe mitral valve insufficiency (ASA status III), the owner declined surgical excision of the tumour. Therefore, microwave ablation (MWA) under local anaesthesia was chosen in order to achieve adequate haemostasis. Both dogs received local anaesthesia around the bleeding mass lesion, and the disintegrated site was microwave‐ablated; dog 1 underwent MWA after blood transfusion to improve the haemorrhagic shock. The ablation site was protected using a non‐adhesive dressing. Scarring of the ulcerated site led to complete haemostasis in both cases. Dog 1 underwent tumorectomy on the 31st hospital day to prevent rebleeding; histopathology results were consistent with mammary adenocarcinoma with the ablation site covered by a capsule structure. To the authors’ knowledge, this is the first case report describing the use of MWA to stop bleeding from mammary tumours in veterinary medicine. MWA is a feasible and potentially effective palliative treatment modality to stop bleeding from disintegrated mammary tumours in dogs under local anaesthesia.

## Graphical abstract

Microwave ablation was performed to control bleeding from a disintegrated mammary tumors in two elderly dogs. Microwave ablation is a feasible and potentially effective palliative treatment modality to stop bleeding from disintegrated mammary tumors in dogs under local anesthesia.

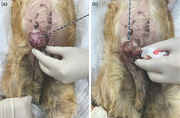

## 1 INTRODUCTION

Mammary tumours – the most frequently found neoplasm in sexually intact female dogs – account for approximately 50% of tumours in female dogs (Priester & Mantel, 1971). The mean age of affected dogs is 10–11 years (range: 2–16 years) (Misdorp, 1988). Histopathologically, nearly 90% tumours arise from epithelial cells, of which nearly 50% are malignant (MacEwen et al., 1985). Furthermore, 50% of tumours have reportedly metastasized by the time of initial examination to primarily lymphogenic sites, including lymph nodes and lungs. Moreover, malignant tumours are often associated with ulceration of the skin covering the tumour and bleeding from these sites; the prognosis of dogs with ulceration is also poorer than that of dogs without ulceration (Hellmén et al., 1993). Additionally, the ulcers can produce offensive odours, which reduce the owner's and patient's quality of life (QOL). The first‐line treatment for mammary tumours with ulceration and bleeding is surgical resection; however, this may not be a preferred option in certain cases, for example when general anaesthesia is difficult owing to the patient's age or poor condition, when benefits of surgery are limited because of metastatic lesions, or when the owner refuses surgery. In such cases, treatment options alternative to surgery are required to control ulceration and bleeding.

Microwave ablation (MWA) is one of the local treatments for malignant tumours, which has been used recently (Alonzo et al., 2015; Ierardi et al., 2015). MWA is a minimally invasive treatment and has anti‐tumour and haemostatic effects (Ke et al., 2015) and is used in various tumour types including mammary gland tumours in human medicine (Imajo et al., 2020; Zhou et al., 2012, 2014).

In this study, MWA was performed on two dogs with persistent bleeding in mammary gland tumours under local anaesthesia. Consequently, immediate ulcer site haemostasis and scarring were observed in both cases, and the owners were adequately satisfied.

## 2 MATERIALS AND METHODS

### 2.1 Animals

#### 2.1.1 Dog 1

**FIGURE 1:**
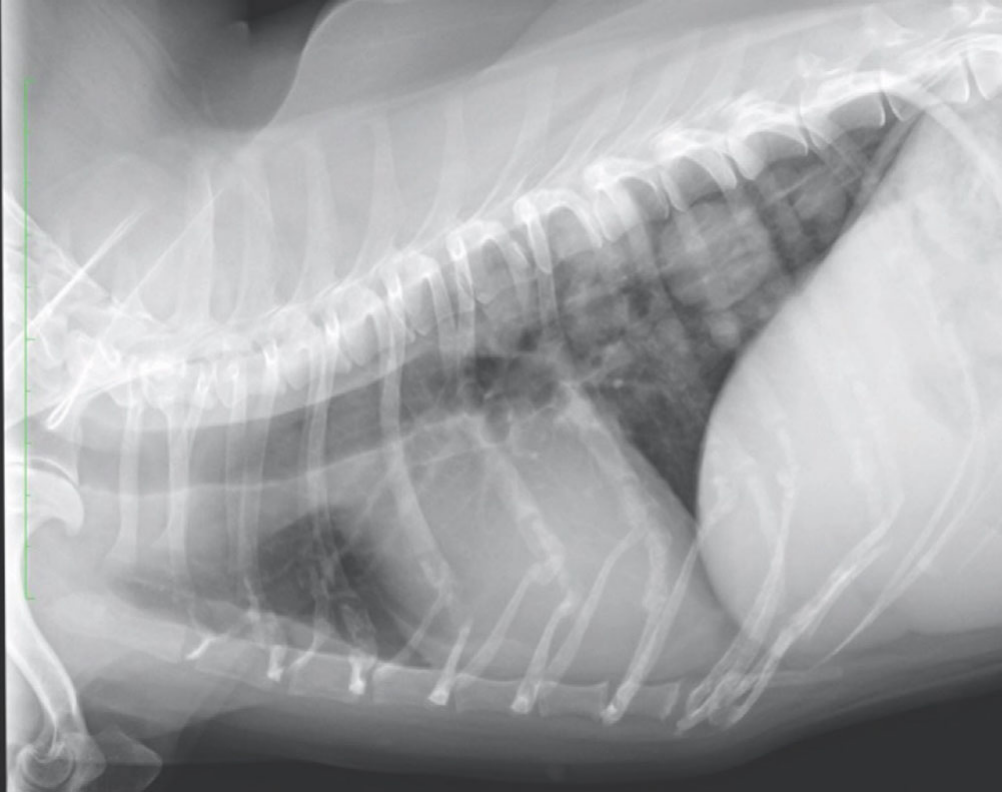
In dog 1, multiple nodular lesions suspected of metastases were observed in the lung field.

**FIGURE 2:**
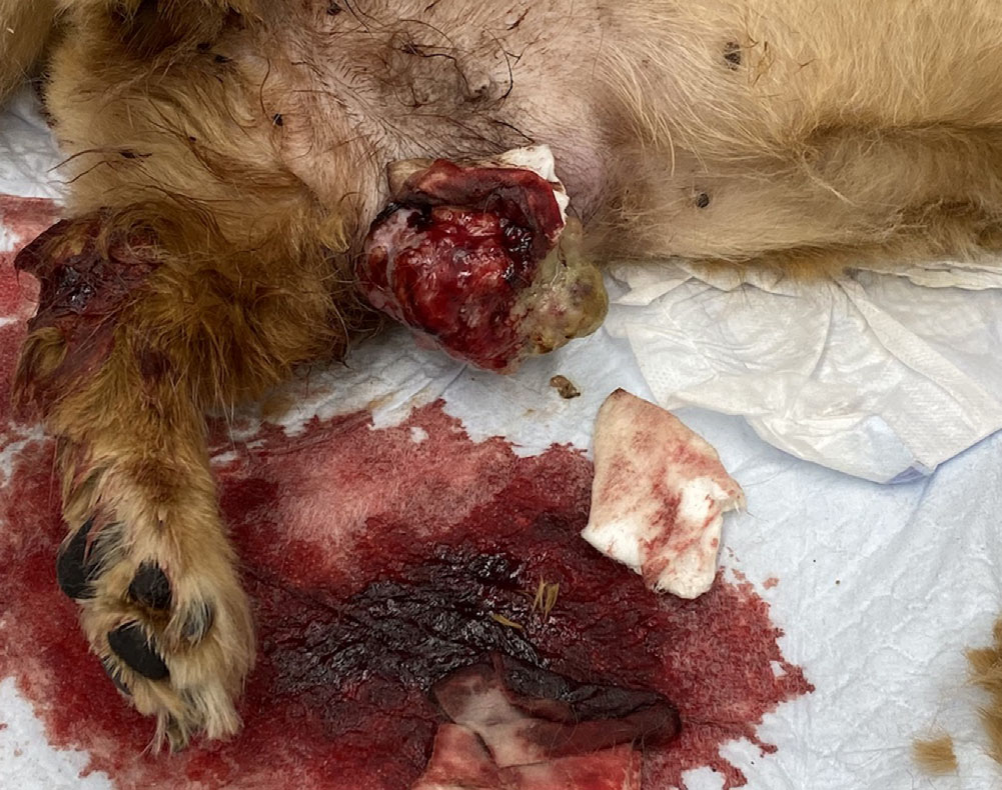
Appearance of the mass in dog 1 at the initial visit. Persistent bleeding that had been difficult to stop was observed.

A 16‐year‐old sexually intact female Miniature Dachshund weighing 5.3 kg was brought to Kawamura Animal hospital with the chief complaint of bleeding from the site of tumour ulceration in a mammary gland. The dog presented with a mass lesion 1 year prior suspected of being a mammary tumour of the fifth left mammary gland; however, the owner refused surgery owing to the dog's advanced age and appearance of metastatic lesions in the lungs. From ∼1 month prior to presentation, the mammary tumour surface had disintegrated; bleeding was controlled with an oral administration of 10 mg/kg q12 h of tranexamic acid and 15 mg/kg q12 h of amoxicillin, and covering the tumour surface with an absorbent pad composed of calcium alginate (Plus Moist Hemosta Pad, Zuiko Medical, Osaka, Japan). An Elizabethan collar was also placed to prevent further trauma to the area. Despite these treatments, intermittent bleeding persisted. The patient was referred to when the bleeding became continuous, due to suspicion for haemorrhagic shock. At the time of arrival, the dog presented with persistent bleeding from the tumour; therefore, examination and treatment were performed while administering compression using a gauze for tumour haemostasis. General physical examination results were as follows: body temperature of 38.2°C, heart rate of 216 bpm, with mildly delayed capillary refill time of 2.0 s, and the visible mucosa was pale. Thoracic radiographs revealed multiple nodular soft tissue lesions suspicious for pulmonary metastasis (Figure 1). A CBC/Chemistry panel revealed anaemia (packed cell volume 18%) and was otherwise unremarkable. The tumour was 6 × 4 × 5 cm^3^ in size; approximately two thirds of the dorsal aspect of the tumour were covered by skin unattached to the tumour, whereas the ventral third of the tumour was adhered to the skin. The surface had disintegrated, and continuous bleeding was occurring from several sites within this area (Figure 2). Due to the patient's advanced age and suspicion of pulmonary metastases, the owner wished to stop the bleeding without surgical intervention with general anaesthesia. After the shock status improved on administering a whole blood transfusion of 100 mL (PCV after transfusion was 26%), MWA was to be performed to control the bleeding under local anaesthesia on the first hospital day.

#### 2.1.2 Dog 2

**FIGURE 3:**
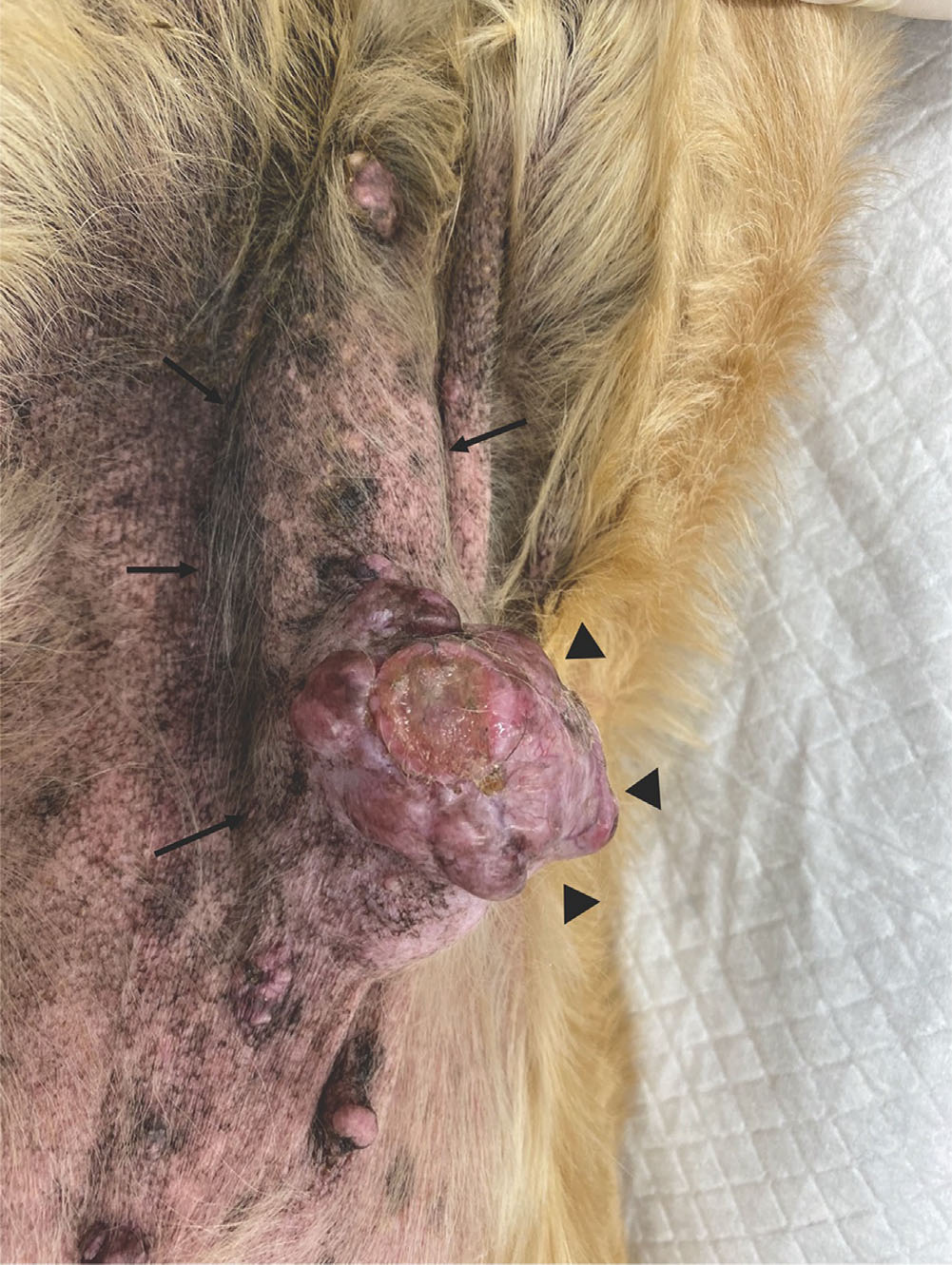
Appearance of the tumour in dog 2 (the arrow indicates the overall image of the tumour, and the arrow head indicates the protruding disintegrated portion).

A 13‐year‐old sexually intact female American Cocker Spaniel weighing 10.7 kg was brought to Kawamura Animal hospital with a chief complaint of an intermittently bleeding mammary gland tumour. She had developed a mass lesion in the right fifth mammary gland, which was intermittently bleeding from the lesion surface; upon consultation for treatment with the local veterinarian, the dog was referred to K hospital. The dog's medical history included the surgical resection of a mass lesion that developed in the left fifth mammary gland approximately 2 years prior by the local veterinarian, at which time the histopathology results revealed mammary adenocarcinoma. Furthermore, ∼1 year ago, the dog developed a perineal hernia with the displacement of the bladder into the perineal area, which necessitated periodic perineal compression for urination. The dog also was diagnosed with mitral valve insufficiency (stage C2 according to the American College of Veterinary Internal Medicine [ACVIM] classification), for which she was receiving enalapril (0.25 mg/kg, twice daily) and pimobendan (0.25 mg/kg, twice daily). Initial physical exam revealed a body temperature of 38.2°C and a heart rate of 186 bpm. Thoracic radiographs revealed cardiac dilatation (vertebral heart scale; VHS = 11.8), and a PCV was performed which revealed mild anaemia (29%). The tumour was 9 × 4 × 6 cm^3^ in size. Two thirds of the dorsal aspect of the tumour were covered by loosely attached skin, whereas the ventral third of the tumour was adhered to the skin. Part of the surface of the attached area had disintegrated and was protruding; within this area, there was intermittent bleeding from several sites (Figure 3). Due to the dog's relatively advanced age, heart disease (ASA status III), and perineal hernia with bladder prolapse, the owner declined surgical resection of the tumour. Alternatively, as the owner wished to stop the intermittent bleeding and avoid relapses, MWA under local anaesthesia was elected to achieve these aims.

### 2.2 MWA

**FIGURE 4:**
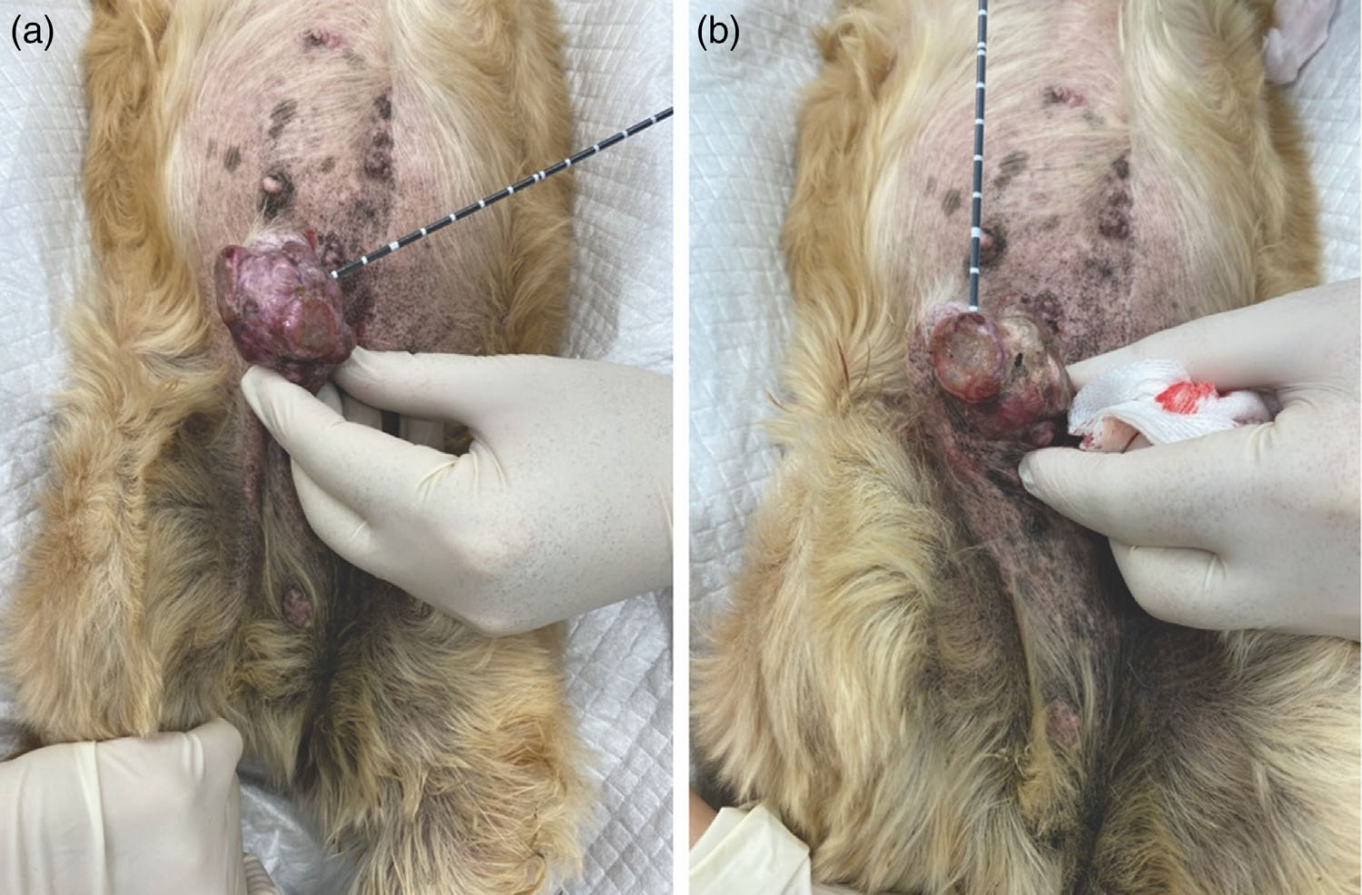
Appearance of the ablated site of dog 2: (a) the first ablation site and (b) the second ablation site. We can confirm that the first ablation site has turned grey.

Preoperatively, a subcutaneous injection of cefovecin (8 mg/kg) was administered as an antibiotic. Midazolam (0.2 mg/kg intramuscular) was used to sedate the patient in a supine position, and the area surrounding the tumour was shaved and disinfected with 0.5% chlorhexidine gluconate. As local anaesthesia, 2% xylocaine solution (0.2 mg/kg) was subcutaneously administered in the area surrounding the tumour to encompass the tumour. The MWA system (Emprint Ablation System, Covidien, Boulder, CO, USA) comprises a generator that produces microwaves, antenna that radially releases microwaves at 2.8 cm from the tip, and a cooling pump. Prior to the procedure, the antenna was attached to the generator; by starting the cooling pump, physiological saline was continuously circulated within the antenna to prevent thermal damage to the antenna. The antenna of the MWA system was directly inserted into the disintegrated ventral third of the tumour in such a manner that the microwave emitter (28 mm from the tip) was entirely embedded within the tumour. Ablation was performed so as to deliver microwave energy to the disintegrated area and the site where the tumour and skin were attached, paying due care not to cause burn injury to the healthy skin. Furthermore, the ablation depth was within the depth of the tumour tissue, and due care was taken such that the thermal damage did not extend to the abdominal wall. The tumour was ablated with an output of 40 W; when the colour of the ablation region turned grey, the output was stopped (Figure 4). To prevent scattering of the ablated tissue because of the popping phenomenon (rupture caused by water boiling) during the procedure, we covered the tumour surface with a gauze moistened with physiological saline and verified the degree of colour change of the ablation region by regularly lifting up the gauze during the procedure. To prevent bleeding from the puncture site, the antenna was slowly removed over 20–30 s while applying 40 W output. This was performed divided into five sites in dog 1 and three sites in dog 2 based on the extent of the disintegrated area. Ablation was performed to overlap sites that had already been ablated such that the entire area was ablated. The time required for one site to completely change colour was 2–3.5 min. We confirmed that bleeding had completely stopped 30 s to 1 min after commencing ablation at sites with continuous preoperative bleeding.

## 3 RESULTS

### 3.1 Dog 1

**FIGURE 5:**
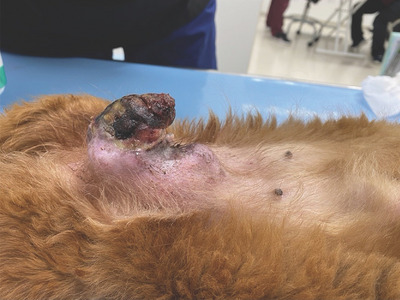
Appearance of the mass in dog 1 on Day 6 following the procedure. A crust had formed on the surface of the ablation site, and the ablated site was reduced in size.

**FIGURE 6:**
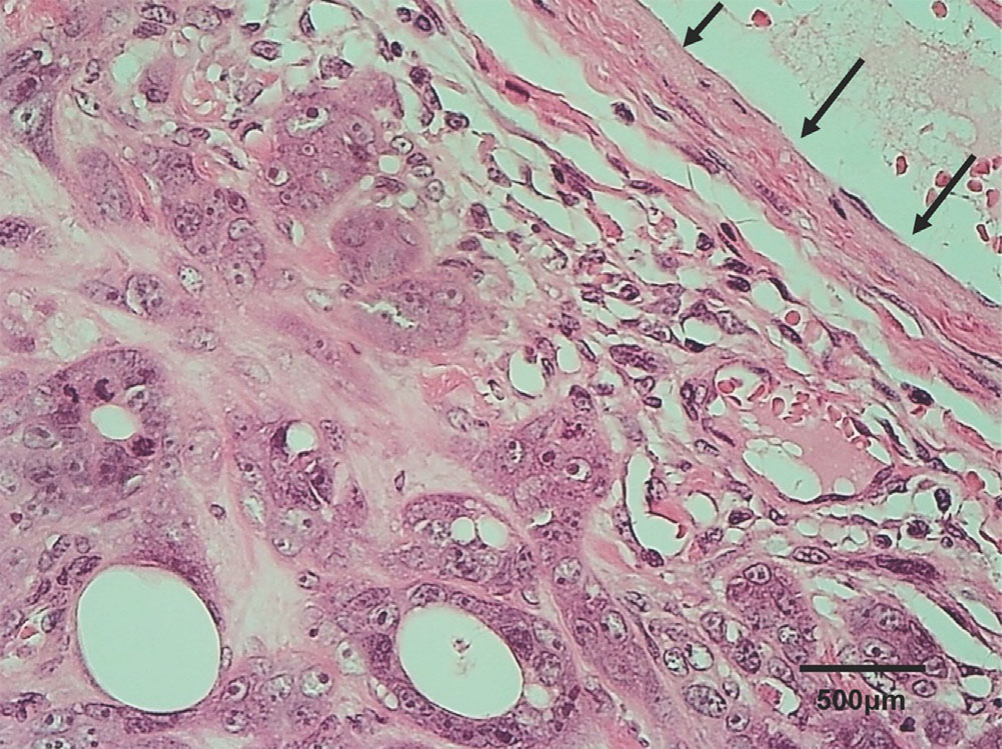
The histopathology results revealed that the tumour was mammary adenocarcinoma, and the ablation site was covered by a capsule structure (arrow).

The dog was discharged on the day following the procedure with a non‐adhesive absorbable dressing (MELOLIN, Smith & Nephew Medical, Watford, England) applied to the ablated site to protect from rubbing by external force. On Day 6 after the procedure, a crust formed on the surface of the ablated site, and we confirmed ablated site shrinkage (Figure 5). As there was no recurrence of bleeding and good general condition was maintained, the owner elected surgical resection of the tumour, whereas the dog's general condition was well enough to avoid the risk for recurrent bleeding. Accordingly, tumorectomy was performed 31 days after MWA. Because the tumorectomy was for a palliative purpose, the tumour was resected at the margin region, and the wound was closed using simple interrupted sutures. The tumour was histopathologically revealed to be mammary adenocarcinoma (Figure 6). The dog died 75 days after initial presentation from respiratory failure because of the metastatic lesions in the lung.

### 3.2 Dog 2

**FIGURE 7:**
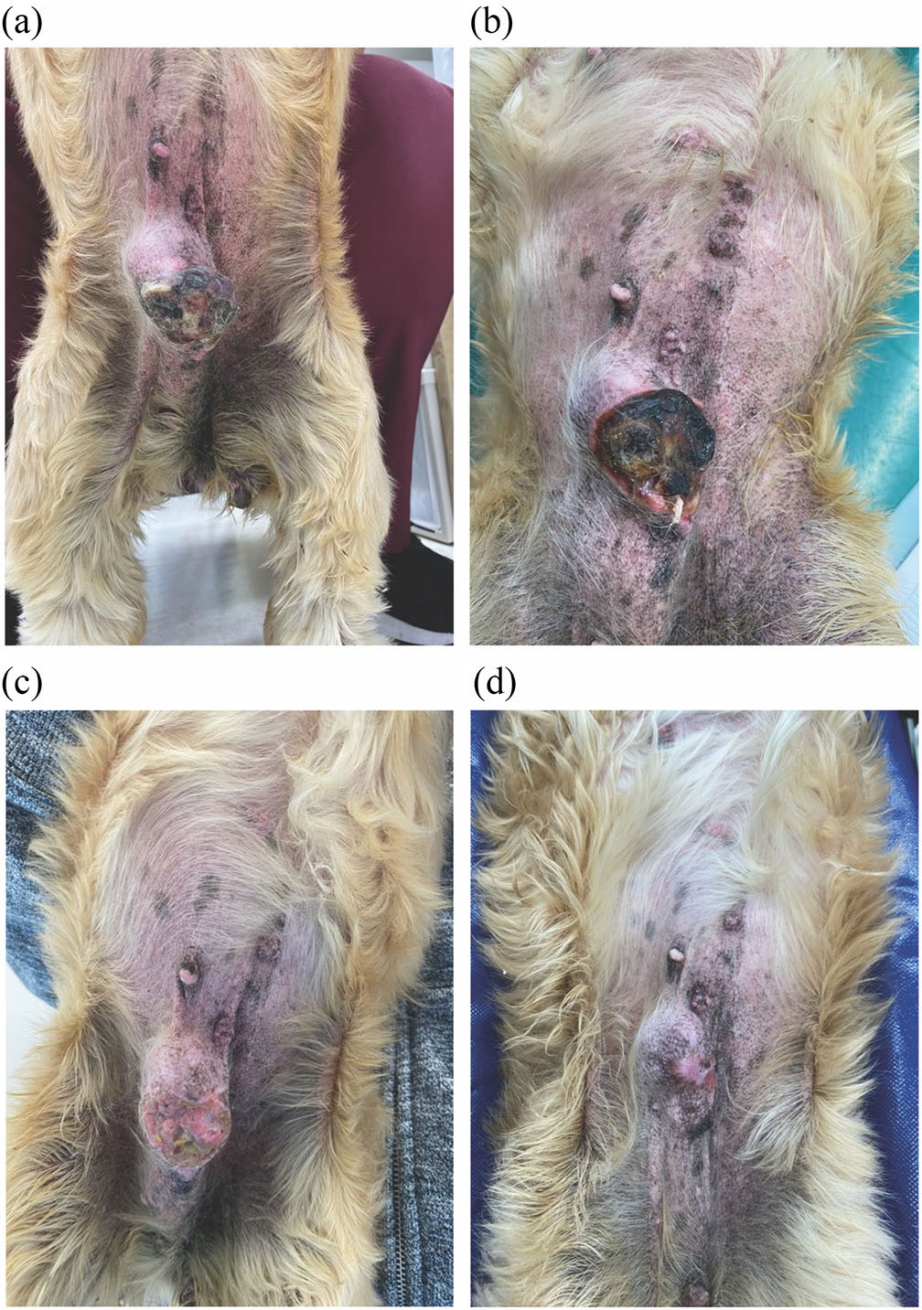
Appearance of the tumour in dog 2 following microwave ablation ((a) Day 2, (b) Day 8, (c) Day 16 and (d) Day 38). After the treatment, a crust quickly formed on the surface of the ablation site, which gradually reduced in size and turned into a scar.

The dog was discharged the day after the procedure with a non‐adhesive absorbable dressing applied to the ablated site to protect from rubbing by external force. On Day 2 following the procedure, a crust formed on the surface of the ablated site. On Day 8, a reduction in the size of the ablation site was confirmed. On Day 16, the crustae came off, and on Day 38, a scar had formed on the site of ablation (Figure 7). At this point, the offensive odour found prior to the procedure had disappeared. As of ∼6 months after the procedure, the dog was alive with no recurrence of bleeding from the tumour and was maintaining good QOL (Figure 7).

## 4 DISCUSSION

This report is possibly the first to describe the use of MWA to treat mammary tumours accompanied by disintegration and bleeding. Immediate haemostasis and scar shrinkage were achieved in the ablated area in both dogs. There were no apparent complications in either dog. Although only two cases are reported here, we confirmed sufficient levels of efficacy in terms of the control of bleeding.

In veterinary medicine, surgical resection is the first‐line treatment to stop bleeding from mammary tumours. However, surgical resection is not always preferred, such as when there is increased risk of general anaesthesia (such as secondary to advanced age or poor overall health), the benefit of surgery is limited due to metastatic disease, or when surgery is refused. In such cases, alternative treatment options are needed to control bleeding. Such options available in human medicine include ablation therapy (Palussière et al., 2012), transcatheter arterial embolization (Aksoy et al., 2016) and chemosurgery using Mohs paste (Trost & Bailin, 2011). Among these treatments, radiofrequency ablation (RFA) is relatively commonly used under local anaesthesia for breast cancer patients who are unsuited to surgery such as elderly patients, and it has started to be used for the radical treatment of early‐stage breast cancer (Palussière et al., 2012). MWA has attracted attention recently because there are many advantages, including large ablation zones, short ablation durations and improved convection profile (Zhou et al., 2014, 2012).

Several studies can be found on the use of Mohs paste as palliative treatment for disintegrated breast tumours in human and veterinary medicine. Mohs paste is an ointment prepared by Mohs et al. in the 1930s to fix tumour tissues; the chemically fixed tumour tissue can be scraped off to reduce exudate and bleeding from the tumour tissue and control pain (Mohs & Guyer, 1941; Trost & Bailin, 2011). Reported problems in using Mohs paste include pain in the affected area where the paste is used and ulceration of the surrounding healthy skin. In veterinary medicine, there is concern on how to prevent a patient from self‐traumatizing the affected area due to the length of time required for the tumour to fixed (minutes to days) (Fukuyama et al., 2016; Hara, 2012). For this reason, a method that fixes the tumour tissues in a short time would be beneficial.

Ablation therapy is broadly divided into RFA and MWA. In these local ablation therapies, a needle called an electrode or antenna is inserted from outside of the body towards the tumour tissue, and heat generated around the needle is used for ablation of the tumour tissue. RFA uses heat (Joule heat) generated because of friction associated with movements of electrons on application of an electric current within a conductor. RFA is limited by the difficulty in predicting the ablation range because the ablation area is oval and blood flow cools the ablation area causing a cooling effect (heat sink). However, MWA uses microwaves generated from an antenna inserted into the body; microwaves cause the rotation of water molecules and produce friction heat, which is applied to ablate tumour tissue. Being less susceptible to the heat sink effect, it is advantageous in that it is easy to control the ablation range, the ablation time is short, and there is no requirement to use a counter electrode plate (Alonzo et al., 2015; Ierardi et al., 2015; Imajo et al., 2020; Yu et al., 2008). Furthermore, the MWA system used in this study circulates a coolant within the antenna portion, thereby avoiding the tissue in the vicinity of the antenna from being exposed to excessive heat and enabling spherical uniform ablation (Alonzo et al., 2015). In veterinary medicine, there have been case reports of MWA performed in malignant lesions in livers, lungs, and bones, in all of which a certain anti‐tumour effect was achieved (Mazzaccari et al., 2017; Oramas et al., 2019; Salyer et al., 2020; Yang et al., 2017). In addition to these practices, in human medicine, ablation therapy has been used to control bleeding from tumours on the body surface that are difficult to surgically resect and has simultaneously brought about rapid haemostasis with the tumour size reduction (Ke et al., 2015). In the present two cases, we applied the haemostatic effect of MWA to the haemostasis of canine mammary gland tumours.

In dog 1, MWA was selected as a means of emergency haemostasis owing to the increased risk of general anaesthesia due to the patient's advanced age (16 years 7 months), and multiple metastatic lesions in the lungs. The dog was anaemic because of intermittent bleeding that was difficult to control despite the use of oral haemostatic agents and absorbent pads containing calcium alginate. Moreover, the bleeding still continued when the dog was brought in for examination. Complete haemostasis was successfully achieved immediately after the procedure and ameliorated the difficult‐to‐control bleeding. Consequently, the dog's general condition improved, and thus surgery to prevent rebleeding could be performed 31 days following ablation. The dog died of respiratory failure because of metastatic lesions in the lungs 75 days following ablation; however, the owner was sufficiently satisfied. In dog 2, the owner had no desire to pursue surgery due to the dog's high anaesthetic risk secondary to mitral valve insufficiency (ACVIM Stage C2) and bladder prolapse secondary to a perineal hernia. We performed MWA according to the owner's desire to control the offensive odour from the ulcerated area and intermittent bleeding. As of ∼2 months after the procedure, bleeding control has been completely achieved; the ablated ventral quarter of the tumour has scarred, and only a small wound presently remains with almost no offensive odour.

In the present two cases, MWA treatment was administered at an output of 40 W, which is used for breast cancer in human medicine (Zhou et al., 2012, 2014) and hepatic neoplasms in veterinary medicine (Yang et al., 2017). In dog 1, in which the ablated tumour tissue was histopathologically examined, we believe that complete coagulative necrosis was achieved by the MWA of the mammary tumour tissue of this case based on the findings of the ablation site. Furthermore, granulation caused by macrophages and other cells was confirmed at the boundary between the ablation site and surrounding area. We believe that this is associated with the fact that the ablation site scars with time and becomes a clean wound as seen in dogs 1 and 2.

MWA for breast cancer in human medicine is performed under local anaesthesia using xylocaine (Zhou et al., 2012, 2014). In both cases of this study, the procedures could be performed under local anaesthesia and under sedation with midazolam. Furthermore, there were no particular procedural complications observed in both dogs included in this study, and both dogs could be discharged the day following the procedure. Complications reported in MWA for breast cancer in human medicine include skin burn injury, bleeding, post‐procedural pain and nipple retraction; the most common complication is skin burn injury with a reported incidence of 19% (Grotenhuis et al., 2013). In this procedure, potential complications are skin burn injury, thermal injury of the abdominal wall, when the ablation range extends to the abdominal wall, and bleeding from the puncture. We believe that it is important to minimize the healthy skin within the ablation range, so that the ablation area stays in the disintegrated site and the site where the tumour and skin are attached. Furthermore, it is important that the depth of the ablation remains within the tumour tissue so that thermal damage does not extend to the abdominal wall. By doing so, we believe that patient's pain can be reduced also. When removing the antenna, we believe that slowly removing the antenna over 20–30 s, whereas applying the 40‐W output is useful to prevent bleeding from the puncture site. When performing under local anaesthesia, pain possibly produced when the ablation range approaches the abdominal wall can pose a problem. This method can be indicated only when there is nonablated tumour tissue between the abdominal wall and the site to be ablated such as disintegration site and bleeding site. When the ablation range and abdominal wall are in proximity, as reported in humans (Wang & Kao, 2015), physiological saline may be injected between the tumour and the structure, such as the abdominal wall, to be protected.

## 5 CONCLUSIONS

We performed MWA under local anaesthesia to control bleeding from a disintegrated mammary tumour in two cases. In both cases, rapid haemostasis was achieved, and the ulcerated tumour tissue scarred. The results of the present two cases suggest that MWA can be performed under local anaesthesia and is potentially effective as palliative treatment to stop bleeding from disintegrated mammary tumours in dogs. We showed the potential of MWA as an effective treatment for patients who are unable to undergo general anaesthesia, for owners who do not wish their dogs to undergo surgery, or for control of temporary bleeding until surgery. Furthermore, aside from mammary tumours, we believe that similar to human studies, MWA can be applied to control bleeding from other body surface tumours that are anatomically difficult to resect and disintegration of such tumours (Ke et al., 2015). A prospective study is required to evaluate the selection of suitable cases and the effectiveness.

## AUTHOR CONTRIBUTIONS

*Project administration; writing – original draft*: Yuta Kawamura. *Writing – review and editing*: Hiroki Itou, Akitomo Kida, Hiroki Sunakawa, Kenji Kawamura.

## CONFLICT OF INTEREST STATEMENT

The authors declare that there were no conflict of interests related to this report.

## ETHICS STATEMENT

The authors confirm that the ethical policies of the journal, as noted on the journal's author guidelines page, have been adhered.

### PEER REVIEW

The peer review history for this article is available at https://publons.com/publon/10.1002/vms3.1089

## Data Availability

The data that support the findings of this study are available from the corresponding author upon reasonable request.
